# Assessing the Organ Dose in Diagnostic Imaging with Digital Tomosynthesis System Using TLD100H Dosimeters

**DOI:** 10.3390/tomography11030032

**Published:** 2025-03-11

**Authors:** Giuseppe Stella, Grazia Asero, Mariajessica Nicotra, Giuliana Candiano, Rosaria Galvagno, Anna Maria Gueli

**Affiliations:** 1 Department of Physics and Astronomy “Ettore Majorana”, University of Catania, Via S. Sofia 64, 95123 Catania, Italy; rosaria.galvagno@phd.unict.it (R.G.); anna.gueli@unict.it (A.M.G.); 2 Azienda Sanitaria Provinciale di Siracusa, P.O. “A. Rizza”, U. O. di Radioterapia, Viale Epipoli 72, 96100 Siracusa, Italy; grazia.asero@asp.sr.it (G.A.); giuliana.candiano@asp.sr.it (G.C.); 3 Specialization School of Medical Physics, Department of Medical and Surgical Sciences and Advanced Technologies “G. F. Ingrassia”, School of Medicine, Via Santa Sofia 78, 95123 Catania, Italy; mariajessica.nicotra@studium.unict.it; 4 Centro Siciliano di Fisica Nucleare e Struttura della Materia (CSFNSM), Viale A. Doria 6, 95125 Catania, Italy

**Keywords:** organ dose, LiF: Mg, Cu, P, radiation dose optimization

## Abstract

Background: Digital tomosynthesis (DTS) is an advanced imaging modality that enhances diagnostic accuracy by offering three-dimensional visualization from two-dimensional projections, which is particularly beneficial in breast and lung imaging. However, this increased imaging capability raises concerns about patient exposure to ionizing radiation. Methods: This study explores the energy and angular dependence of thermoluminescent dosimeters (TLDs), specifically TLD100H, to improve the accuracy of organ dose assessment during DTS. Using a comprehensive experimental approach, organ doses were measured in both DTS and traditional RX modes. Results: The results showed lung doses of approximately 3.21 mGy for the left lung and 3.32 mGy for the right lung during DTS, aligning with the existing literature. In contrast, the RX mode yielded significantly lower lung doses of 0.33 mGy. The heart dose during DTS was measured at 2.81 mGy, corroborating findings from similar studies. Conclusions: These results reinforce the reliability of TLD100H dosimetry in assessing radiation exposure and highlight the need for optimizing imaging protocols to minimize doses. Overall, this study contributes to the ongoing dialogue on enhancing patient safety in diagnostic imaging and advocates for collaboration among medical physicists, radiologists, and technologists to establish best practices.

## 1. Introduction

Digital tomosynthesis (DTS) is an innovative imaging technology that bridges the gap between traditional radiography and computed tomography (CT), offering three-dimensional (3D) visualization at a significantly lower dose compared to CT. Originally developed for breast imaging, DTS has increasingly been applied in various medical fields, including thoracic and musculoskeletal imaging [1,2,3,4,5,6,7,8]. One of the key advantages of DTS is its ability to acquire multiple low-dose projection images at different angles, which are then reconstructed into slice images. This approach improves the visibility of subtle lesions by reducing the overlap of anatomical structures, a limitation often seen in traditional 2D radiographs.

In thoracic imaging, DTS has proven particularly effective for detecting pulmonary nodules, providing better depth resolution and reducing anatomical clutter compared to conventional chest radiography. Studies by Zachrisson et al. [1] and Quaia et al. [3] have shown that DTS significantly improves the detection of lung nodules, with detection rates comparable to those of CT but at much lower radiation doses. However, despite the reduced dose compared to CT, DTS typically delivers higher radiation doses than conventional radiography, raising concerns about radiation exposure, especially in patients requiring repeated imaging, such as those with chronic lung diseases or a high risk of lung cancer.

Optimization of the radiation dose remains a crucial issue in clinical practice. Recent studies have addressed organ dose concerns, focusing on minimizing exposure while maintaining diagnostic accuracy. For instance, Mohd Norsuddin et al. [9] established local diagnostic reference levels (DRLs) for digital breast tomosynthesis (DBT) in Malaysia, revealing that the average glandular dose (AGD) for DBT is higher than that for full-field digital mammography (FFDM). This finding underscores the need for continuous monitoring of radiation exposure, especially as DBT usage increases.

In musculoskeletal imaging, DTS has shown significant advantages in evaluating complex fractures and orthopedic conditions, particularly in cases involving metallic implants. Its ability to achieve a high in-plane resolution and perform weight-bearing exams is particularly valuable for diagnosing joint instability and fracture healing. While CT remains the gold standard for detailed bone imaging, DTS offers a viable, lower-dose alternative for specific diagnostic tasks [10].

Efforts to optimize imaging protocols and reduce radiation exposure, especially for pediatric patients and other high-risk groups, are ongoing [11]. Advanced dosimetric techniques are crucial for providing real-time feedback to radiologists, thereby optimizing radiation exposure levels. Moreover, the development of a database of organ dose coefficients, as seen in the study of Sharma et al. [12], who used Monte Carlo simulations to generate patient-specific dose coefficients, may be instrumental in further refining protocols and improving individual dose estimation.

The current study aims to assess the organ dose using DTS in comparison to conventional radiography, with a particular focus on lung and heart exposure. By utilizing TLD100H dosimeters [13,14,15], this study seeks to contribute to the ongoing discussion on optimizing DTS protocols, ensuring a balance between diagnostic efficacy and patient safety.

## 2. Materials and Methods

### 2.1. Dosimeters

Luminescence emissions from a high-sensitivity LiF: Mg, Cu, P dosimeter, commercially named TLD100H [16,17], were performed using a Riso TL/OSL-DA-15 reader (Tokyo, Japan). A 9635QA photomultiplier tube combined with a Hoya U340 optical filter (Shinjuku, Tokyo) were used for detection of luminescence emissions. All measurements were conducted in a nitrogen atmosphere with a constant heating rate of 1 °C/s from Room Temperature (RT) to 240 °C and a preheating temperature of 190 °C for 5 s.

Annealing was performed with two heating cycles at 240 °C for 60 s. To minimize fading effects, luminescence measurements were carried out 12 h after irradiation [13]. To analyze TL signals and extract kinetic parameters, experimental glow peaks, and the luminescence contributions of different peaks, glow-curve deconvolution (GCD) was applied using the First Order kinetics equation [18]:

(1) $$I\left(T\right)=I_{m}\exp\left[1+\frac{E}{kT}\;\frac{T-T_{m}}{T_{m}}-\frac{T^{2}}{{T^{2}}_{m}}\times \exp\left[\frac{E}{kT}\;\frac{T-T_{m}}{T_{m}}\right]\left(1-∆\right)-{∆}_{m}\right]$$

where I represents the glow-peak intensity, $I_{m}$ is the intensity at the peak maximum, $T_{m}$ (K) is the temperature at the peak maximum intensity, T is the stimulation temperature during readout, E (eV) is the activation energy, and k (eV K^−1^) is the Boltzmann constant, with $∆=2kT/E,\;{∆}_{m}=2kT_{m}/E$.

The intensity at the peak maximum ($I_{m}$) and the temperature at the peak maximum intensity ($T_{m}$) depend on the frequency factor *s* (s^−1^) and the heating rate *β* (K/s) according to the following equations:

(2) $$s=\frac{\beta E}{kT_{m}^{2}}\exp\;\left(\frac{E}{kT_{m}}\right)$$

(3) $$I_{m}=n_{0}\frac{\beta E}{kT_{m}^{2}}\exp\left[-\left(1-{∆}_{m}\right)\right]$$

The quality of fit was tested with the Figure Of Merit (FOM) of Balian and Eddy (1977) [19]. For each fit, a FOM value of about 2% was found, which usually indicates a satisfactory fit.

During the laboratory measurement, room lighting was provided by Illford DL10 lamps equipped with Ilford #902 filters in order to minimize the effects of optical bleaching [20].

### 2.2. Energy and Angular Dependence Studies

For the study of energy and angular dependence, the TLDs were irradiated using the Agfa DR800 system (https://agfaradiologysolutions.com/digital-tomosynthesis/; accessed on 8 January 2025), which is equipped with a digital tomosynthesis system. This system combines 3D multi-slice reconstruction with a fast and efficient general radiology workflow by varying the acquisition angle between 20 and 40 degrees.

**Figure 1:**
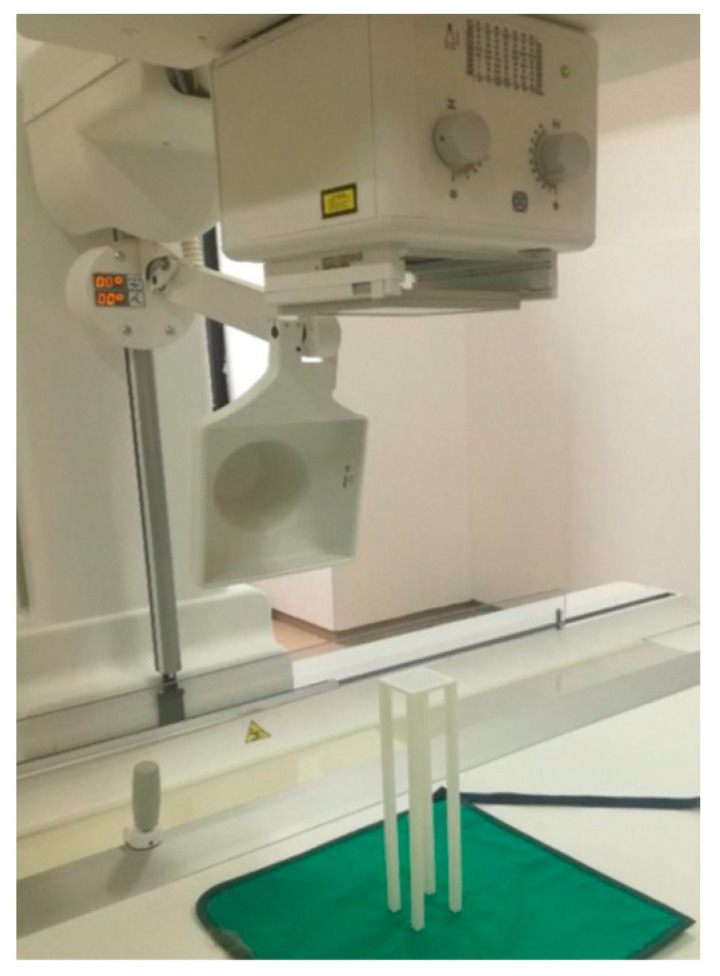
In-house printed PLA (PolyLactic Acid) phantom.

The TLDs were positioned on an in-house printed PLA (PolyLactic Acid) phantom with dimensions of 30 cm in height, a base of 5 cm × 5 cm, and an infill density of 20%. This phantom was designed using Autodesk Inventor Professional 2022 CAD software and manufactured with a Raise3D Pro2 Plus printer (Irvine, CA, USA). The PLA surface supporting the TLDs has a thickness of less than 1 mm to minimize backscattering effects (Figure 1).

For the energy dependence study, 21 TLDs, grouped in sets of three, were irradiated in a fixed tube with an SSD of 75 cm at different beam qualities (HVL): 2.4 mmAl (70 kVp); 2.8 mmAl (80 kVp); 3.2 mmAl (90 kVp); and 3.6 mmAl (100 kVp).

The tube output at varying beam qualities was measured using a Piranha meter (RTI black Piranha meter, else solutions) (Table 1).

Table 2 presents the output values in milliampere-seconds per milliGray (mAs/mGy) for different kV settings, specifically at different beam qualities (HVL).

For the angular dependence study, 15 TLDs, divided into groups of three, were irradiated at different tube angles, as shown in Table 3. The dose values obtained for each angle were normalized to an SSD of 75 cm by accounting for the dose variation with the square of the distance.

### 2.3. Organ Dose Calculation

**Figure 2:**
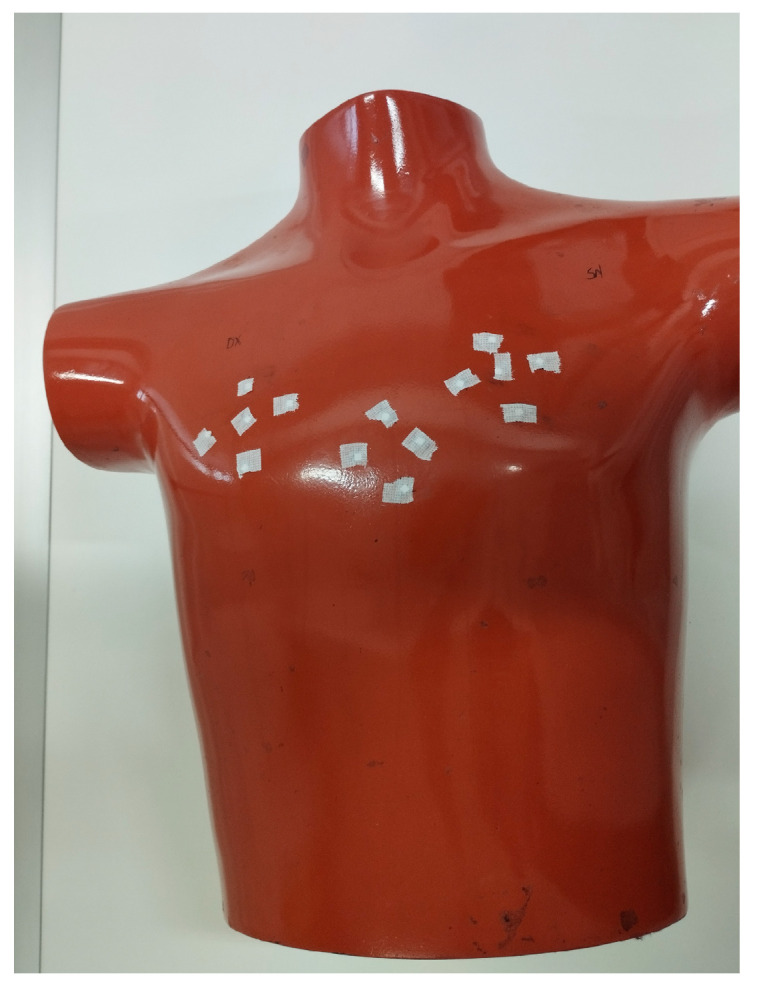
Positioning of the thermoluminescent dosimeters (TLDs) on the skin of the THOR phantom, indicating specific locations for lung (left and right) and heart dose measurements during both digital tomosynthesis (DTS) and traditional fixed-tube (RX) imaging modes.

For the experimental measurement of the organ dose, the AP diagnostic protocol was used in two different modes: traditional fixed tube (RX) and digital tomosynthesis (DTS). Fourteen TLDs were placed on the skin of a THOR anthropomorphic phantom, specifically 5 on the left lung, 5 on the right lung, and 4 on the heart (Figure 2).

Table 4 shows the parameters used for DTS and RX acquisition modes.

In particular, the organs of interest are the skin, lungs and heart. To calculate the organ dose, the American Association of Physicists in Medicine (AAPM) TG-61 [21] formalism was employed. The dose absorbed by a specific tissue type was determined via the following equation:

(4) $$Dose\;\left(mGy\right)={\left(K_{air}^{TLD}\right)}^{air}N_{x}{\left[{\left(\frac{{\bar{\mu }}_{en}}{\rho }\right)}_{air}^{w}\right]}_{air}B_{w}C_{w}^{tissue}$$

where ${\left(K_{air}^{TLD}\right)}^{air}$ represents the air kerma obtained from the analytical relation between TL intensity (by TLD) response and dose; $N_{x}$ is the air kerma calibration coefficient for a particular beam quality; $C_{w}^{tissue}$ represents the free-in-air ratio of mass energy-absorption coefficients of biological tissue to water, which is dependent on the beam quality, as indicated by the half-value layer (HVL) measurement; ${\left[{\left(\frac{{\bar{\mu }}_{en}}{\rho }\right)}_{air}^{w}\right]}_{air}$ is the ratio of the average mass energy-absorption coefficients for water to air and free in air, and to convert air kerma to water kerma as a function of HVL (mm Al); $B_{w}$ is the backscatter factor that accounts for the effect of scattering owing to the water phantom. In this study, considering the parameters reported in Table 5, the following coefficient were used: $N_{x}=1$; ${\left[{\left(\frac{{\bar{\mu }}_{en}}{\rho }\right)}_{air}^{w}\right]}_{air}=1.025;\;B_{w}=1.419$; $C_{w}^{skin}=0.929$; $C_{w}^{lung}=1.042$; and $C_{w}^{heart}=1.030$.

## 3. Results and Discussion

### 3.1. Deconvolution Procedures

Using the fixed-tube configuration of 3.6 mmAl (100 kVp) with an SSD of 75 cm and HVL = 3.6 mmAl (100 kVp), five TLDs were irradiated in order to identify the correct fitting parameters for the deconvolution process.

**Figure 3:**
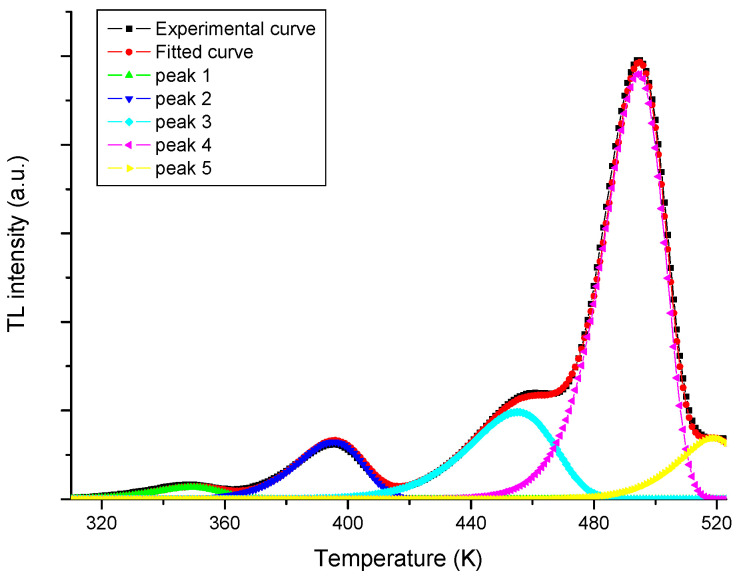
A glow-curve deconvolution procedure showing the luminescence contributions of TL peaks 1, 2, 3, 4, and 5.

Figure 3 presents an example of the experimental TL data and the luminescence contributions of the four individual peaks obtained through the deconvolution procedures using Equation (1). The fitting procedures were performed using in-house-developed software.

Table 5 shows the average results obtained for the five TLDs in terms of the peak temperature (T_m_) in K and trap depth (E) in eV, and the frequency factor s (s^−1^), with an average FOM of approximately (1.90 ± 0.05) %.

The useful peak for dosimetry, due to its signal stability and repeatability characteristics, is peak 4. For peak 5, the trap depth is not reported as it is affected by the background and the maximum temperature cutoff [13,14,15].

As mentioned in Section 2.1, in order to eliminate the unstable thermoluminescence component and isolate the contribution from useful peak 4 for dosimetry, pre-heating at 190 °C was applied after irradiation and before the TL reading was taken.

**Figure 4:**
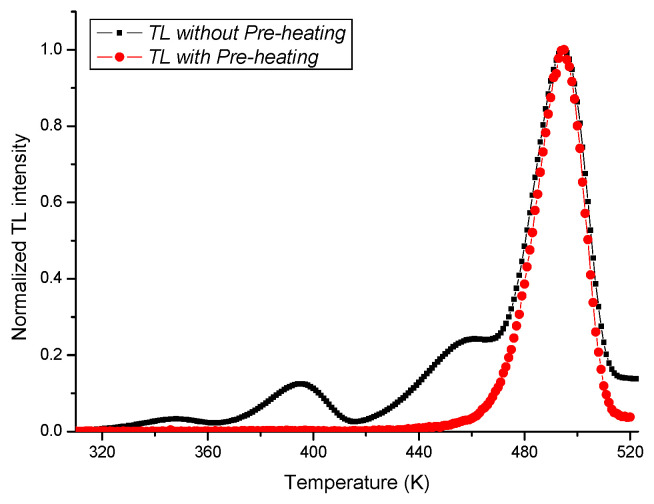
Comparison between the TL curve without pre-heating and that after pre-heating.

Figure 4 shows the comparison between the TL curve without pre-heating and that after pre-heating.

### 3.2. Energy and Angular Dependence Studies

Understanding the energy and angular dependence of thermoluminescent dosimeters (TLDs) is crucial for accurate dosimetric assessments in digital tomosynthesis (DTS) procedures. All of the TLDs were irradiated with the same dose for each beam quality (HVL). From the obtained responses, normalization factors were calculated with respect to the average value of the luminescent response. Each normalization factor was then associated with each individual TLD to correct the response, due to differences in mass and sensitivity.

**Figure 5:**
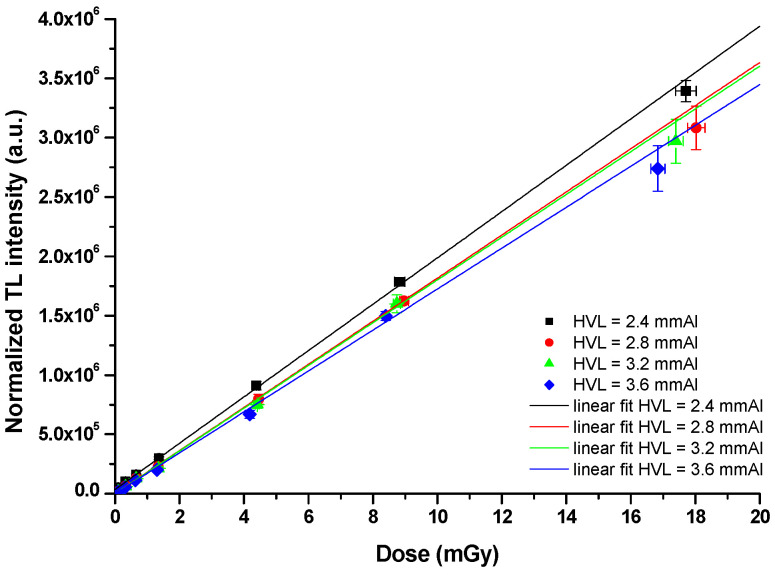
Dose–response relationship obtained from the thermoluminescent dosimeters (TLDs) across different beam qualities in DTS procedure.

Figure 5 presents the dose–response curve for various tube voltages, highlighting the relationship between beam quality and the resultant dose recorded by TLDs. This relationship is fundamental, as it informs the calibration processes necessary for ensuring that the dosimeters accurately reflect the radiation doses delivered to patients.

The dose–response data depicted in Figure 5 indicate significant variations in response based on the energy of the X-ray beam. Table 6 summarizes the calculated dosimetric percentage differences (matrix elements) that arise from comparing the various calibration curves shown in Figure 4. A pronounced energy dependence of the TLDs is evident, particularly outside the beam quality range (HVL) of 2.8 mmAl to 3.2 mmAl. This observation underscores the importance of using appropriate calibration curves that match the specific beam qualities employed in clinical settings.

These percentage differences reflect how the calibration curves can affect the dosimetric readings based on beam energy. Notably, the values indicate that when calibration is not accurately matched to the beam quality, dosimetric inaccuracies can exceed 12%, potentially leading to significant implications for patient safety and treatment efficacy.

**Figure 6:**
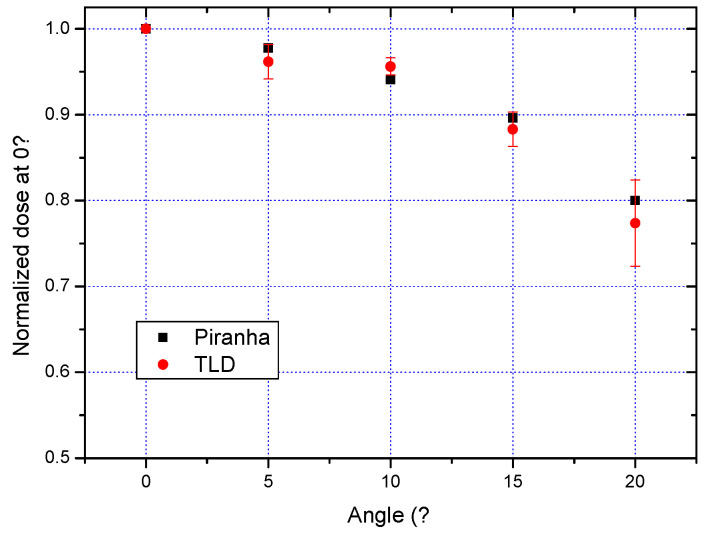
Normalized dose values at a 0° angle, comparing results from both the Piranha system and the TLDs. The data, normalized to a source-to-surface distance (SSD) of 75 cm, illustrate the variation in dose delivery, with a notable 25% difference observed at an angle of 20°.

Additionally, Figure 6 illustrates the trend in the normalized dose at 0°, measured using both the Piranha system and TLDs. The normalization process accounts for a source-to-surface distance (SSD) of 75 cm, allowing for a more standardized comparison of dose delivery across measurement systems. The data reveal a substantial variation, with TLDs showing approximately a 25% difference at an angle of 20°. This discrepancy is crucial for interpreting dosimetric results and emphasizes the importance of considering angular dependence in dose assessments. The alignment of TLD measurements with those from the Piranha system reinforces the validity of the findings, providing confidence in the reliability of TLDs as a dosimetric tool within DTS.

These results highlight the necessity for ongoing calibration and validation of dosimetric systems, particularly in complex imaging modalities like DTS where variations in energy and angle can significantly impact upon dose delivery. Understanding these dependencies not only enhances the precision of dosimetry but also supports the overarching goal of optimizing patient safety and imaging quality in clinical practice.

### 3.3. Organ Dose Calculation

Table 7 presents a comprehensive summary of organ doses measured using thermoluminescent dosimeters (TLDs) positioned on the skin of a THOR anthropomorphic phantom during both digital tomosynthesis (DTS) and traditional fixed-tube radiography (RX) modes. These data are critical for understanding the differential radiation exposure associated with each imaging technique, especially in terms of protecting sensitive organs.

The findings clearly illustrate that during DTS, the organ doses are significantly higher compared to traditional RX. For the left lung (Lung SX), the dose recorded during DTS was 3.32 mGy, while the right lung (Lung Dx) received a dose of 3.21 mGy. In contrast, lung doses during the RX mode were markedly lower at 0.33 mGy for the left lung and 0.31 mGy for the right lung. This stark difference underscores the effectiveness of DTS in delivering high-quality imaging while still maintaining a reasonable level of radiation exposure, especially when imaging the lungs, which are often a primary area of concern in chest imaging.

The heart dose recorded during DTS was 2.81 mGy, reflecting a controlled exposure level associated with advanced imaging techniques. In comparison, the heart dose during RX mode was notably lower at 0.27 mGy. This information is particularly relevant for patient populations at a higher risk of radiation-induced complications, such as those with underlying cardiovascular conditions.

In this study, the doses for the lung, heart, and skin were calculated using the American Association of Physicists in Medicine (AAPM) TG-61 formalism [21], which involves the application of specific tissue-to-water mass energy-absorption coefficient ratios, denoted as $C_{w}^{tissue}$, for each organ. These ratios are used to convert the dose calculated for a water phantom to the corresponding dose in the biological tissue.

However, it is important to clarify that the doses calculated in this study represent the entrance dose to the organ, specifically the dose at the surface of a water phantom, and not directly the actual absorbed organ dose itself. According to the AAPM TG-61, the coefficients $C_{w}^{tissue}$ are used to calculate the dose at the surface of a phantom made of water (or an equivalent material), not the biological tissue directly.

These coefficients account for the differences in energy absorption between water and biological tissues (such as lung, heart, and skin). The dose calculated for the phantom (water) is then adjusted by these coefficients to approximate the entrance dose to the biological tissue.

Therefore, the values calculated in this study correspond to entrance doses to the organ rather than the actual absorbed dose within the tissue. The entrance dose is the dose received at the surface of the organ, not considering the full interaction of the radiation within the tissue, which would require more complex modeling of the energy deposition and scattering processes within the tissue.

The exposure angles and the material differences between the water-equivalent and anthropomorphic phantoms, particularly in the thoracic region with lung-equivalent material, could introduce uncertainties in the dosimetric calculations.

Uncertainties must be taken into account. Studies by other authors, such as that of the authors in [22], find that when using an anthropomorphic phantom, the average backscatter factors are around 1.286. This introduces an uncertainty of approximately 10% in our dosimetric evaluations. This uncertainty should be added to the experimental uncertainty obtained (Table 7).

Additional uncertainties of 5% could be introduced by the actual variation in field size due to gantry rotation and the angle dependence of TLDs. This consideration was made by evaluating a maximum diameter variation of about 5 cm [21], as well as the average angular variation in the attenuated dose (Figure 6).

Overall, these results emphasize the capacity of TLD100H dosimeters to provide accurate assessments of organ doses, offering valuable insight into the radiation burden associated with different imaging modalities. The considerable differences in doses highlight the necessity for continuous optimization of imaging protocols to enhance patient safety while still achieving diagnostic efficacy. By carefully considering the trade-offs between image quality and radiation exposure, clinicians can make more informed decisions that prioritize patient health and minimize potential risks. Such analyses not only guide best practices in imaging but also reinforce the importance of ongoing education and training for radiology professionals in the context of radiation safety.

## 4. Conclusions

This study has provided valuable insights into the organ doses associated with digital tomosynthesis (DTS), highlighting the technique’s potential for enhanced diagnostic accuracy while emphasizing the importance of radiation safety. The use of TLD100H dosimeters allowed for the consistent and reliable measurements of organ doses, with the left lung receiving a dose of approximately 3.21 mGy and the right lung receiving 3.32 mGy during DTS. The heart dose was measured at 2.81 mGy, aligning with results from phantom studies and supporting the notion that advanced imaging techniques can effectively manage radiation exposure with careful protocol optimization.

The preliminary characterization of the TLDs in this study underscored the importance of accounting for the energy and angular dependence of TLDs in dosimetric measurements. The consistency between the TLD data and the Piranha system data further validated the reliability of the measurements, reinforcing the accuracy of the dose assessments made. Overall, the results corroborate previous findings, which indicate that while DTS delivers a significantly lower dose than CT, it imparts a higher dose than conventional radiography.

In conclusion, DTS represents a significant advancement in diagnostic imaging by offering improved accuracy in detecting subtle lesions while maintaining a relatively low radiation dose compared to CT. However, it is important to note that despite its benefits, DTS still results in a higher dose than standard radiography, necessitating continued efforts to optimize imaging protocols and minimize radiation exposure. As the technology continues to evolve, it will be essential to refine dosimetric practices, integrate these advancements into clinical routines, and ensure that radiation safety remains a priority. By doing so, DTS can continue to serve as a safe and effective diagnostic tool, particularly in applications where precision and low radiation exposure are critical.

Ultimately, while DTS offers clear advantages in terms of diagnostic performance and dose reduction compared to CT, further refinement of imaging protocols and ongoing assessment of organ dose exposure will be essential to maximizing its clinical utility. By combining advances in dosimetric techniques with optimized imaging practices, it will be possible to strike a balance between diagnostic accuracy and patient safety, particularly for high-risk or vulnerable populations.

## Figures and Tables

**Table 1:** The table displays the milliampere-seconds (mAs) values measured for TLD numbers 1–21 at different beam qualities, illustrating the relationship between TLD number and exposure across different voltage settings in DTS.

	2.4 mmAl	2.8 mmAl	3.2 mmAl	3.6 mmAl
TLD Number	mAs	mAs	mAs	mAs
1–3	1.2	1.0	0.8	0.6
4–6	4.0	3.2	2.5	2.0
7–9	8.0	6.3	5.0	4.0
10–12	16.0	12.5	10.0	8.0
13–15	50.0	40.0	32.0	25.0
16–18	100.0	80.0	63.0	50.0
19–21	200.0	160.0	125.0	100.0

**Table 2:** Output values in milliampere-seconds per milliGray (mAs/mGy) for different kV settings, specifically at different beam qualities (HVL), providing insight into the relationship between tube voltage and radiation output in DTS.

HVL	Output (mAs/mGy)
2.4 mmAl	0.08 ± 0.01
2.8 mmAl	0.11 ± 0.01
3.2 mmAl	0.13 ± 0.01
3.6 mmAl	0.16 ± 0.01

**Table 3:** Milliampere-seconds (mAs) and corresponding angles (°) for different tube angles at HVL = 2.8 mmAl, measured with a source-to-surface distance (SSD) of 75 cm, highlighting the variation in dose delivery based on angular positioning during DTS process.

HVL	mAs	Angle (°)	SSD (cm)
2.8 mmAl	12.5	0	75
		5	77
		10	77
		15	80
		20	82

**Table 4:** Settings used during the traditional fixed-tube (RX) and digital tomosynthesis (DTS) modes; key parameters such as kV, half-value layer (HVL), milliampere-seconds (mAs), field size, source-to-surface distance (SSD), tube angle, and the number of projections, providing insight into the differences in imaging protocols.

	RX Mode	DTS Mode
kV	100	100
HVL	3.6 mmAl	3.6 mmAl
mAs	1.63	14.33
Field	40 cm × 40 cm	40 cm × 40 cm
SSD	80 cm	80 cm
Angle tube	Fixed zero	Sweep angle 15°
Number of projections	1	67

**Table 5:** Fit parameters of the four peaks obtained by the deconvolution procedure.

Peak Number	*T_m_* (K)	*E* (eV)	*s* (s^−1^)
1	347 ± 3	0.95 ± 0.03	5.48 × 10^12^
2	394 ± 3	1.07 ± 0.04	3.73 × 10^12^
3	455 ± 4	1.32 ± 0.05	3.09 × 10^13^
4	495 ± 4	2.24 ± 0.06	7.20 × 10^21^
5	521 ± 5	-	-

**Table 6:** Calculated dosimetric percentage differences for various calibration curves. The values in mmAl refer to the HVLs of the beam used.

	2.4 mmAl	2.8 mmAl	3.2 mmAl
**2.8 mmAl**	12.3%	-	-
**3.2 mmAl**	12.5%	0.2%	-
**3.6 mmAl**	17.5%	4.7%	4.5%

**Table 7:** Organ doses recorded using thermoluminescent dosimeters (TLDs) placed on the skin of a THOR anthropomorphic phantom; doses for the left lung, right lung, and heart for both imaging methods, highlighting the differences in radiation exposure associated with each technique.

TLDs Position	Method	Skin Dose (mGy)	Lung Dose (mGy)	Heart Dose (mGy)
Lung SX	DTS	2.96 [2.77, 3.30]	3.32 [3.10, 3.46]	
	RX	0.29 [0.24, 0.40]	0.33 [0.27, 0.45]	
Lung Dx	DTS	2.86 [2.47, 3.16]	3.21 [2.77, 3.55]	
	RX	0.27 [0.23, 0.30]	0.31 [0.26, 0.34]	
Heart	DTS	2.81 [2.61, 3.14]		3.11 [2.90, 3.49]
	RX	0.25 [0.22, 0.26]		0.27 [0.25, 0.29]

## Data Availability

The original contributions presented in the study are included in the article; further inquiries can be directed to the corresponding author.
